# A postoperative scoring system for post-hepatectomy early recurrence of colorectal liver metastases

**DOI:** 10.18632/oncotarget.20934

**Published:** 2017-09-15

**Authors:** Rui Mao, Jian-Jun Zhao, Xin-Yu Bi, Ye-Fan Zhang, Zhi-Yu Li, Jian-Guo Zhou, Xiao-Long Wu, Chen Xiao, Hong Zhao, Jian-Qiang Cai

**Affiliations:** ^1^ Department of Hepatobiliary Surgery, National Cancer Center/Cancer Hospital, Chinese Academy of Medical Sciences and Peking Union Medical College, Beijing, 100021, China

**Keywords:** colorectal liver metastases, hepatectomy, early recurrence, risk factors, salvage treatment

## Abstract

The aims of this study were to assess early recurrence predictive factors and elucidate the best early recurrence management. 255 patients with colorectal liver metastases (CRLM) who underwent hepatectomy were retrospectively analyzed. A total of 87 patients (34.1%) developed early recurrence, defined as recurrence that occurred within 6 months after resection. Multivariate analysis showed that preoperative carcino-embryonic antigen (CEA) level ≥ 30 ng/ml, primary tumor lymphovascular invasion (LVI), number of metastases ≥ 4, R1 resection and initially unresectable disease were independent predictors of early recurrence. A predictive scoring system for early recurrence was created by incorporating these factors, and this system showed good discrimination (concordance index of 0.78). In early recurrent patients who underwent salvage treatment, those with 0–2 risk factors demonstrated a significantly longer median survival after recurrence than patients with 3–5 risk factors (33.4 months vs. 20.2 months, *p* = 0.001). For patients who underwent chemotherapy alone, the median survival after recurrence between two groups was comparable (18.3 months vs. 22.6 months, *p* = 0.926). Multivariate analysis revealed that primary tumor lymph node metastases (HR = 1.96, *p* = 0.032), early recurrence (HR = 1.67, *p* = 0.045), salvage treatment for recurrence (HR = 0.47, *p* = 0.002) and predictive scores for early recurrence (HR = 1.39, *p* = 0.004) were independent factors for survival in patients with recurrence. In patients with early recurrence, bilobar distribution of metastases (HR = 2.05, *p* = 0.025) and salvage treatment for recurrence (HR = 0.46, *p* = 0.019) were independent factors for survival. In conclusion, we developed a predictive model that is a very useful tool for determining both the likelihood of early recurrence and the necessity for salvage treatment.

## INTRODUCTION

Liver is the most frequent site for metastasis from colorectal cancer, with more than 50% of patients developing hepatic metastases during the course of the disease [1]. Liver resection, combined with modern chemotherapy, is considered the standard treatment for patients with resectable CRLM [2–3]. In selected patients, 5-year survival can approach 50% after resection of liver metastases [4]. However, recurrence occurs in 50%–75% of the patients after surgery [5–6], thus remaining a major problem. In particular, the correlation between a short time to recurrence and reduced survival has been demonstrated in published series [7–12]. Hence, active surveillance, earlier adjuvant chemotherapy and intensive chemotherapy regimen may be required for patients with high risk of early recurrence after liver resection for CRLM. Whereas, prognostic factors of early recurrence are not completely clear.

The treatment of recurrence after initial resection is highly varied, including salvage treatment like second surgery [13], percutaneous radiofrequency ablation (RFA) [14], and stereotactic body radiotherapy (SBRT) [15], systematic chemotherapy or a combination of these modalities. When technically feasible, patients with recurrence are always candidates for salvage treatment, in order to increase the chance of long-term survival [16]. However, the indication for salvage therapy of recurrent disease is quite ambiguous. The current treatment strategy is largely based on technical issue: whether the recurrent lesions are anatomically favorable for loco-regional treatment, while other clinicopathological factors are rarely concerned. Early recurrence is often regarded as a marker of aggressive tumor biology, whether salvage treatment could work effectively in patients with early recurrence is largely unknown.

The aim of this study was to identify predictive factors for early recurrence, defined as recurrence within 6 months of CRLM resection and to evaluate the prognosis after early recurrence. We also sought to identify which patients could benefit from salvage treatment after early recurrence.

## RESULTS

### Clinicopathological features

The study population comprised 255 patients (156 males and 99 females) with median age of 56. The clinicopathologic features of the patients in the study are displayed in Table 1. 196 patients (76.9%) presented with synchronous disease. 126 patients (49.4%) had a solitary liver metastases, with a median of 2 lesions, and a maximum of 15 lesions. The median diameter of hepatic lesion was 2.8 cm, with 123 patients (48.2%) had a lesion larger than 3 cm. Bilobar distribution of metastases was observed in 82 patients (32.2%). Preoperative chemotherapy was administered in 174 patients (68.2%), among which 144 were the initially unresectable (56.5%). Simultaneous resection of colorectal and hepatic tumor was performed in 152 patients (59.6%). The most common surgical resection performed was nonanatomic hepatectomy (*n* = 199, 78%), followed by anatomic plus nonanatomic (*n* = 36, 14.1%) and anatomic resection (*n* = 20, 7.9%). 71 patients (27.8%) had margin invasion on pathological examination. Major complications were reported in 24 patients (9.4%). The overall follow-up in this study sample was 28.6 months (range: 5.3–99.9 months).

**Table 1:** Clinicopathologic characteristics of patients with or without early recurrence

	All Patients *n* = 255 (%)	Early recurrence group *n* = 87 (%)	Early recurrence-free group*n* = 168 (%)	*p*
Male sex, *n* (%)	156 (61.1)	49 (56.3)	107 (63.7)	0.252
Age, (range)	56 (28–79)	57 (32–79)	55 (28–78)	0.413
Age ≥ 60, *n* (%)	135 (52.9)	44 (50.6)	91 (54.2)	0.586
Preoperative CEA, (range), ng/ml	8.4 (1.4–1503.0)	12.2 (2.0–593.9)	6.0 (1.4–1503.0)	0.000
Preoperative CEA ≥ 30 ng/ml, *n* (%)	64 (25.1)	34 (39.1)	30 (17.9)	0.000
Primary site, *n*(%)				
Colo*n*	138 (54.1)	47 (54.0)	91 (54.2)	0.983
Left hemicolo*n*	213 (83.5)	73 (83.9)	140 (83.3)	0.907
T3-4, *n* (%)	242 (94.9)	84 (96.6)	158 (94.0)	0.574
Lymphovascular invasion, *n* (%)	74 (29.0)	41 (47.1)	33 (19.6)	0.000
Perineural invasion, *n* (%)	81 (31.8)	34 (39.1)	47 (28.0)	0.071
Node-positive primary tumor,*n* (%)	177 (69.4)	71 (81.6)	106 (63.1)	0.002
Synchronous metastasis, *n*(%)	196 (76.9)	70 (80.5)	126 (75.0)	0.327
Initial unresectability,*n* (%)	144 (56.5)	60 (69.0)	84 (50.0)	0.004
Number of metastases, (range)	2 (1–15)	3 (1–15)	1 (1–15)	0.000
≥4 liver metastases, *n*(%)	113 (81)	65 (82)	48 (79)	0.593
Bilobar distribution, *n*(%)	82 (32.2)	44 (50.6)	38 (22.6)	0.000
Diameter of metastases, (range), cm	2.8 (0.5–20)	3.0 (1–16)	2.7 (0.5–20)	0.429
Diameter of metastases ≥ 3 cm, *n* (%)	123 (48.2)	46 (52.9)	77 (45.8)	0.286
High-moderate differentiation,*n* (%)	199 (78.0)	62 (71.3)	137 (81.5)	0.06
Preoperative chemotherapy, *n*(%)	177 (69.4)	65 (74.7)	112 (66.7)	0.186
Simultaneous primary tumor resection, *n* (%)	152 (59.6)	53 (60.9)	99 (58.9)	0.759
Nonanatomical resection, *n* (%)	199 (78.0)	70 (80.5)	129 (76.8)	0.502
Laparoscopic hepatectomy, *n*(%)	18 (7.1)	4 (4.6)	14 (8.3)	0.27
Positive surgical margins, *n* (%)	71 (27.8)	37 (42.5)	34 (20.2)	0.000
Intraopeartive transfusion, *n*(%)	45 (17.6)	16 (18.4)	29 (17.3)	0.839
Major complications, *n*(%)	24 (9.4)	13 (14.9)	11 (6.5)	0.03

### Recurrence and survival

Recurrences were detected in 166 (65.1%) patients, including 87 early recurrences (recurrence within 6 months) and 79 late recurrences (recurrence after 6 months). Patients with early recurrence were more likely to develop intrahepatic recurrences than those with late recurrence (65.5% vs. 48.1%; *p* = 0.024), while extrahepatic recurrences were less common in patients with early recurrence (18.4% vs. 40.5%; *p* = 0.002). Simultaneous intrahepatic and extrahepatic recurrences rates were similar (16.1% vs. 11.4%; *p* = 0.381) (Table 2).

**Table 2:** Recurrence pattern and salvage treatment for recurrence

	Early recurrence *n* = 87 (%)	Late recurrence *n* = 79 (%)	*p*
Intrahepatic recurrence	57 (65.5%)	38 (48.1%)	0.024
Extrahepatic recurrence	16 (18.4%)	32 (40.5%)	0.002
Lung	8	17	
Peritoneum	3	4	
Lymph node	1	6	
Multiple sites	2	3	
Others	2	2	
Intra+Extrahepatic recurrence	14 (16.1%)	9 (11.4%)	0.381
Liver+Lung	10	5	
Liver+Lymph node	3	3	
Liver+Peritoneum	1	0	
Liver+others	0	1	
Salvage treatment for recurrence	35 (40.2%)	37 (46.8%)	0.391
Radiofrequency ablation	25	22	
Second resection	6	13	
TACE	2	2	
SBRT	2	0	

TACE = transcatheter arterial chemoembolization; SBRT = stereotactic body radiotherapy.

**Figure 1:**
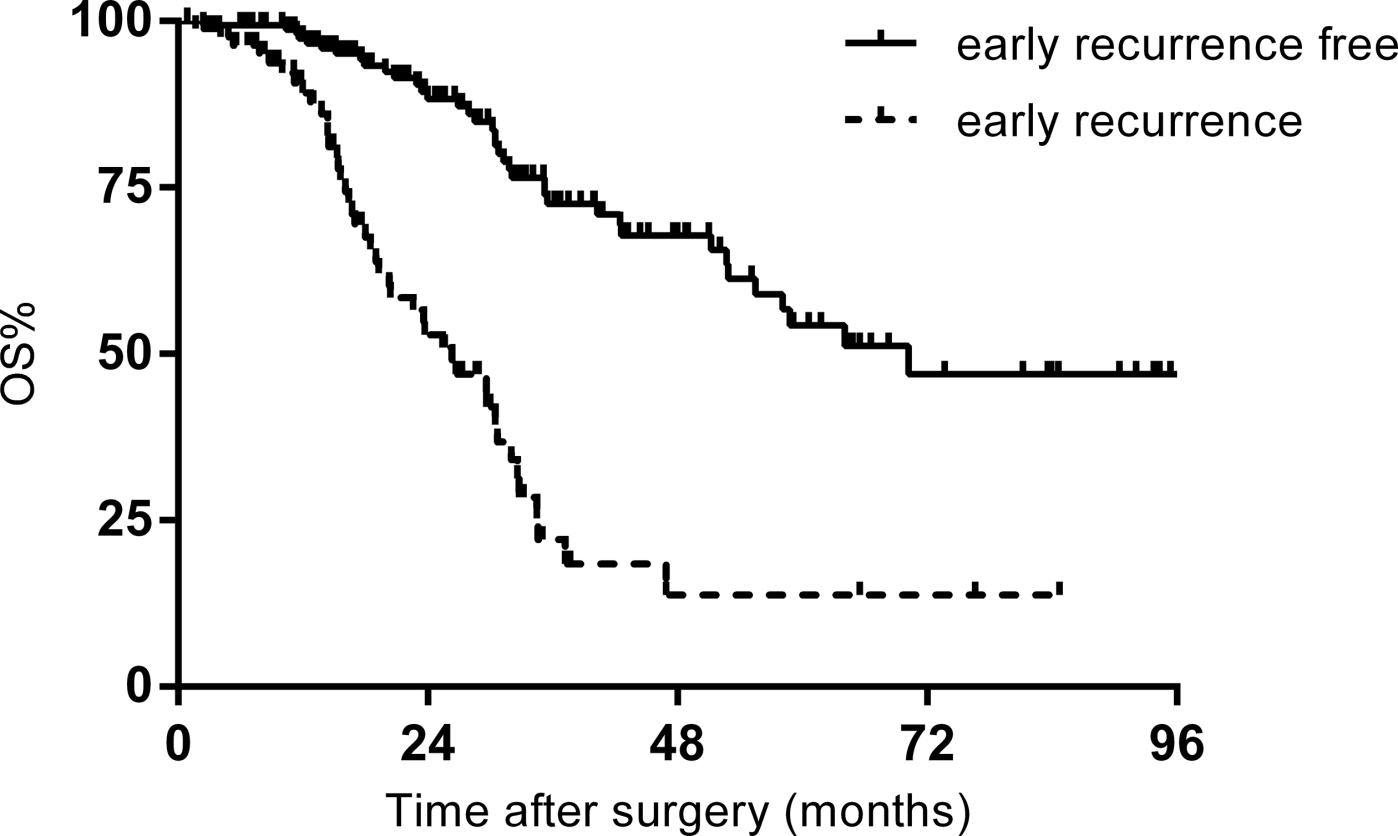
Overall survival (OS) of patients with and without early recurrence

The included patients were divided into an early recurrence group (*n* = 87) and an early recurrence-free group (*n* = 168) composed of patients without evidence of recurrence within 6 months after surgery. Median survival for the whole study population was 51.2 months. The 1-, 3-, and 5-year OS rates were 95.6%, 56.2%, and 41.0%, respectively. Survival was significantly worse in the early recurrence group compared with the early recurrence-free group (5-year OS: 54.3% vs. 11.8 %, *p* = 0.000) (Figure 1).

### Predictive factors for early recurrence

Table 1 summarizes the differences in clinicopathological features of the early recurrence and early recurrence-free group. There were more patients with preoperative CEA ≥ 30ng/ml (53.1% vs 27.7%; *p* = 0.000), primary tumor lymphovascular invasion (LVI) (55.4% vs 25.0%; *p* = 0.000), lymph node metastases (40.1% vs 20.5%; *p* = 0.000), initially unresectable disease (41.7% vs 24.3%; *p* = 0.004), the number of metastases ≥ 4 (70.2% vs 26.3%; *p* = 0.000), bilobar metastases (63.2% vs 27.4%; *p* = 0.000), R1 resection (52.1% vs 27.2%; *p* = 0.000) and major complications (54.2% vs 32.0%; *p* = 0.03) who developed early recurrence. Multivariate analysis showed that preoperative CEA ≥ 30 ng/ml (HR = 2.41; 95% CI = 1.23–4.73; *p* = 0.01), primary tumor LVI (HR = 2.54; 95% CI = 1.35–4.77; *p* = 0.004), the number of metastases ≥ 4 (HR = 3.05; 95% CI = 1.52–6.09; *p* = 0.002), R1 resection (HR = 2.22; 95% CI = 1.17–4.20; *p* = 0.015) and initially unresectable disease (HR = 1.93; 95% CI = 1.02–3.65; *p* = 0.045) were independent predictors of early recurrence after curative resection of CRLM. According to the Hosmer-Lemeshow test, the *P*-value was 0.220 (*P* > 0.05), which indicates the fit of the logistic regression model.

Based on the number of independent predictors each patient had, a scoring system for the prediction of early recurrence was generated (range 0–5 points), and the estimated risk of early recurrence according to the score is shown in Table 3. This scoring system showed a good discrimination ability (concordance index of 0.78, 95% CI = 0.74–0.82). Subgroup Analyses in patients with preoperative chemotherapy obtained consistent results (concordance index of 0.79, 95% CI = 0.73–0.87).

**Table 3:** Total points and estimated risk of early recurrence

Total points	Estimated risk of early recurrence	Early recurrence-free
0	4.1%	95.9%
1	24.7%	75.3%
2	41.9%	58.1%
3	53.8%	46.2%
4	81.3%	18.7%
5	100%	0%

### Early recurrence treatment

**Figure 2:**
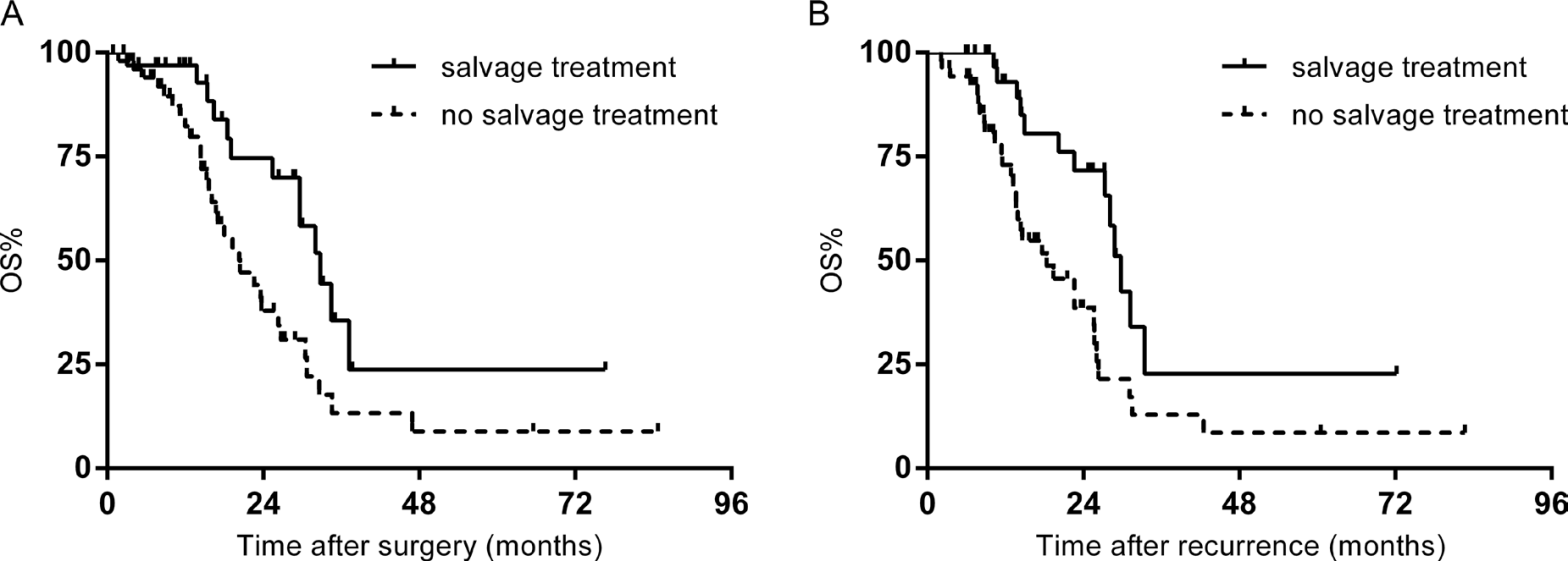
(**A**) OS of patients with early recurrence stratified by treatment. (**B**) OS after recurrence in patients with early recurrence stratified by treatment.

Treatment after recurrence was displayed in Table 2. 35 patients (40.2%) with early recurrences and 37 patients (46.8%) with late recurrences received salvage treatments. There was no significant difference in rates of recurrence beyond salvage treatment in the two cohort of patients (59.2% vs. 53.2%, *p* = 0.391). In the early recurrence group, 3-year OS rate following first hepatectomy in patients with salvage treatment was 35.5%, versus 13.3% of patients that received chemotherapy alone (*p* = 0.013) (Figure 2A). After a median follow-up of 23.8 months after recurrence, 3-year OS rate was 22.6% in patients treated with salvage treatment, compared with 12.9% of patients who underwent chemotherapy alone (*p* = 0.008) (Figure 2B).

**Figure 3:**
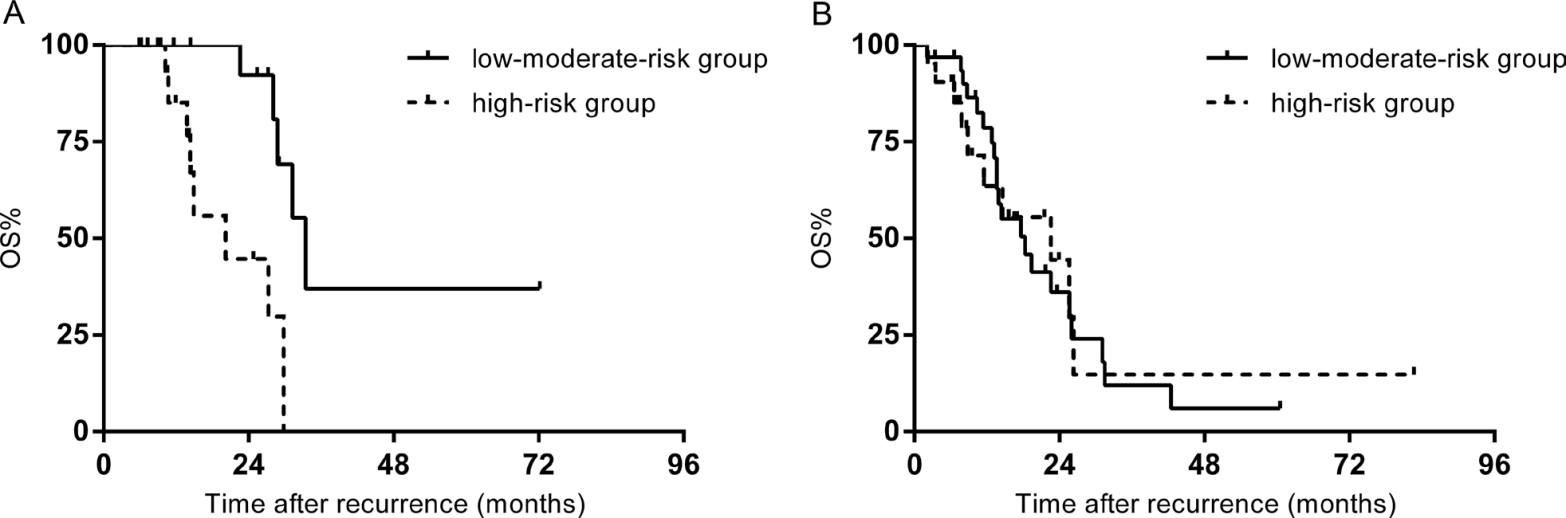
(**A**) OS after recurrence of patients who underwent salvage treatment stratified by risks for early recurrence. (**B**) OS after recurrence in patients who underwent palliative chemotherapy stratified by risks for early recurrence.

Patients with early recurrence were further divided into two subgroups according to the scoring system (low-moderate-risk group: 0–2 points; high-risk group: 3–5 points). No statistically significant difference was found in median survival time after recurrence between two groups (26.0 months vs. 22.6 months, *p* = 0.154). In patients who underwent salvage treatment, the low-moderate-risk group demonstrated a significantly longer median survival after recurrence than the high-risk group (33.4 months vs. 20.2 months, *p* = 0.002) (Figure 3A). For those salvage treatment was not indicated, the median survival after recurrence in the low-moderate-risk group was 18.3 months, comparable to the 22.6 months in the high-risk group (*p* = 0.926) (Figure 3B).

### Prognostic factors for patients with recurrence

For the entire cohort of patients with recurrent disease after liver resection, multivariate analysis revealed primary tumor lymph node metastases (HR = 1.96; 95% CI = 1.06–3.61; *p* = 0.032), early recurrence (HR = 1.67; 95% CI = 1.01–2.74; *p* = 0.045), salvage treatment for recurrence (HR = 0.47; 95% CI = 0.29–0.75; *p* = 0.002) and predictive scores for early recurrence (HR = 1.39; 95% CI = 1.11–1.74; *p* = 0.004) were independent prognostic factors for survival. In early recurrence group, bilobar distribution of metastases (HR = 2.05; 95% CI = 1.09–3.84; *p* = 0.025) and salvage treatment for recurrence (HR = 0.46; 95% CI = 0.24–0.88; *p* = 0.019) were independent factors for survival. In patients with late recurrence, predictive scores for early recurrence (HR = 1.69; 95% CI = 1.17–2.43; *p* = 0.005) and salvage treatment for recurrence (HR = 0.41; 95% CI = 0.21–0.84; *p* = 0.014) were independent factors.

## DISCUSSION

Numerous studies have confirmed the possibility of long-term survival after hepatectomy for CRLM. However, early recurrence after initial hepatectomy remains a major concern. In current study, 34.1% of patients experienced recurrence within 6 months after surgery. Five factors were identified to be associated with early recurrence after hepatectomy: preoperative CEA ≥ 30 ng/ml, primary tumor LVI, the number of metastases ≥ 4, R1 resection and initially unresectable disease. Moreover, when patients were stratified according to the number of risk factors, the efficacy of salvage treatment was significantly influenced.

The cutoff value for early recurrence varied in different series, ranging from 6 months to 2 years [7–12, 17–18]. Current study defined early recurrence as a relapsed disease within 6 months, which is the most commonly adopted definition. Consistent with previous reports, OS in the early recurrence group was significantly worse, and early recurrence was identified to be an independent unfavorable prognostic factor. Although it was hypothesized that patients who developed early recurrence could benefit little from hepatectomy [9], our results showed these patients had a median survival of 26.3 months, better than the reported survival of patients who receive chemotherapy alone [19–20]. This result suggested that hepatectomy is justified in patients with high risk for early recurrence.

The most common site of early recurrence was intrahepatic recurrence. Meanwhile, the number of metastases ≥ 4 and R1 resection were identified as independent predictors for early recurrence, suggesting that missed lesions and residual microscopic tumor cells left in the first hepatectomy both contribute to early intrahepatic recurrence. Resection margin status could also serve as a symbol of biological behavior of a tumor, because R0 resection is more likely to be achieved when patients have a favorable tumor biology. Moreover, the clinical significance of surgical margin is further complicated by preoperative chemotherapy. Indeed, several studies have demonstrated that tumor biology rather than R1 resection was independently associated with survival, and the long-term outcome benefit conferred by R0 resection disappeared in the setting of modern chemotherapy [21–23]. Since detailed intrahepatic recurrence pattern was not analyzed, it is not clear whether recurrence at the resection margin or new intrahepatic metastases, which respectively represent technical and biological reasons, account most for early recurrence in patients with positive margin. After all, R1 resection showed independent association with early recurrence while adjusting for other clinical and pathological factors, in the context of preoperative chemotherapy was given to 68.2% of the patients. Hence, microscopically negative margins should always be attempted when it is safe and feasible.

Resectability rate of CRLM has been increased to 10–40% with the development conversion chemotherapy [24–25]. Whereas, although the converted patients have a similar OS after surgery compared to patients with initially resectable disease, they tended to have a shorter disease free interval, which could be as short as 3.2 months [10, 26–27]. The current study reaffirms the finding, and further identified initial unresecbility as an independent factor for early recurrence, implying that these patients could benefit less from the hepatectomy. The correlation with early recurrence and initial unresectability may be attributed to delayed adjuvant chemotherapy due to higher incidence of postoperative complications, and higher R1 rates caused by more complex surgical procedure. Therefore, If patients with initially unresectable CRLM are present with other risk factors for early recurrence, they may benefit less from the conversion strategy. Surgical indication for initially unresectable CLM with these factor should be considered cautiously. Of cause, definition of unresectability is subjective and differs among doctors and hospitals. In our institution, a general definition of unresectability is applied and all controversial cases are discussed in MDT, thus a relatively standardized criteria is guaranteed.

Pathological factors of the primary tumor is recognized to help discern the secondary lesions. Among which, LVI has been demonstrated to be a poor prognostic factor for patients with CRLM after hepatectomy. Previous studies have confirmed that patients with LVI positive primary colorectal tumors had a unfavorable OS and PFS compared to those without LVI in their primary colorectal tumor [28–29]. Our results have also shown that patients with LVI were more likely to develop early recurrence, which reflects the importance of pathological details of primary tumor in assessing prognosis after liver resection. Moreover, 50–60% of patients with CRC develop metachronous liver metastases after radical resection of colorectal carcinoma, thus the pathological factors of the primary tumor may help surgeons discern a group of patients who will benefit more from hepatectomy.

This study identified that short interval between first surgery and recurrence is not a determinant factor for salvage treatment. The salvage treatment rates were similar, and long-term outcome was significantly improved by salvage treatment compared with palliative chemotherapy in patients with early recurrence. Even though one might argue the outcome may be a result of a generally favorable tumor biology in patients who received salvage treatment, salvage treatment remained an independent predictor for survival in multivariate analysis. Hence, early recurrence is not a contraindication for salvage treatment. The major concern is when recurrent disease is localized and salvage treatment is indicated, could every patient with early recurrence benefit? In current study, the survival benefit conferred by aggressive treatment disappeared in patients with more than 2 risk factors for early recurrence. Indeed, the long-term results of salvage treatment in this cohort of patients was significantly worse than that of patients with 0–2 risk factors. Moreover, the survival time after salvage treatment was not significantly different from that after palliative chemotherapy (20.2 months vs 22.6 months). The result suggested the therapeutic effect of salvage treatment could be offset by unfavorable tumor biology in a subgroup of patients with high risk for early recurrence. Instead of controlling local recurrence, salvage treatment possibly facilitate rapid progression and postoperative complications in these patients, which in turn decrease survival. Thus, routine application of salvage treatment in patients with early recurrence whenever possible is questionable, and other factors apart from the location of recurrent disease should be considered on an individual basis to choose an appropriate treatment for early recurrence.

Based on the five risk factors, a scoring system was created. This predictive model included both biological and technical factors, and may thus be useful for identifying patients with high risk for early recurrence. Such patients are appropriate for more close surveillance, but they may not be appropriate candidates for intensive treatment when recurrence occurs. As far as we know, our predictive model is the first for early recurrence after hepatectomy for CRLM. Although most patients in the study received preoperative chemotherapy that could affect PFS [30], subgroup analyses of patients who underwent preoperative chemotherapy showed a good discrimination ability. This individual prediction of the patient's risk of early relapse may assist oncologists in deciding an optimal treatment strategy for individual patients with CRLM. The major drawback is that the scoring system has only been validated internally and further external validation is warranted. Besides, criteria for unresectability varied from institutions, which would unavoidably result in bias. This study has some other limitations. First, this is a retrospective study with a relatively small sample size. Second, protective effect of adjuvant chemotherapy was not considered in current study, because we aim to propose a algorithm based on tumor biology and surgery-related factors, in order to identify a group of patients for earlier and more intensive adjuvant chemotherapy to delay recurrence.

## MATERIALS AND METHODS

### Inclusion and exclusion criteria

Consecutive patients who underwent surgery for CRLM at Department of hepatobiliary surgery, Cancer Hospital, Chinese Academy of Medical Sciences between January 1, 2007, and November 30, 2015, were identified from our prospective institutional database. Inclusion criteria were: (1) patients who underwent hepatectomy for curative intent; (2) histologically proven colorectal adenocarcinoma liver metastases; (3) a follow-up more than 6 months. Exclusion criteria were: (1) extrahepatic metastases detected on preoperative imaging or during surgery; (2) R2 resection; (3) combined with RFA; (4) postoperative deaths (noncancer-related 90-day mortality); (5) a history of prior hepatectomy for CRLM.

### Perioperative management

All patients were evaluated preoperatively, including CEA levels; abdominopelvic computed tomography (CT) and magnetic resonance imaging (MRI); and chest radiography or chest CT to determine the disease stage. Preoperative chemotherapy was recommended to patients with initially unresectable CRLM, or to patients with multiple high risk factors: synchronous metastases, ≥ 4 hepatic lesions, primary tumor invading nearby tissues/organs and imaged mesenteric nodal disease. The definitions of unresectability were as follows: multiple liver metastases that required resection of more than 70% of non-tumor liver for removal of all tumors, tumors invading all three hepatic veins, tumors invading both the left and right branches of the hepatic artery or portal vein, and unresectable extrahepatic metastases. Chemotherapy was composed of a combination of 5-fluorouracil/capecitabine and oxaliplatin/irinotecan with or without bevacizumab and cetuximab. Tumor response was assessed according to the Response Evaluation Criteria In Solid Tumors criteria (RECIST, version 1.1) every two cycles. All imaging studies were reviewed by at least two independent radiologists until the final conclusion was drawn. Surgery with curative intent was performed if hepatic lesions were considered treatable with hepatectomy. The decision to undertake surgery for controversial cases was reached by consensus of a multidisciplinary team (MDT) including surgeons, oncologists and radiologists.

During surgery, the peritoneal cavity was inspected to eliminate previously undetected extrahepatic disease. Manual liver palpation and intraoperative ultrasound was used to rule out occult lesions and confirm the number, size and location of the liver metastases. The principle of surgery was to remove all detectable lesions with a tumor-free margin. All specimens were subjected to histologic evaluation to confirm the pathological diagnosis, number and size of liver lesions, and the width of surgical margin. In case of multiple liver metastases, the diameter of the largest lesion was defined as the final size, and the closest margin was recorded. R1 resection was defined with a distance from the metastasis edge to the transection line of less than 1 mm.

Postoperative complications were graded according to the Clavien system and major complications were defined as any complication of grade III or IV. After discharge, adjuvant chemotherapy was recommended to most patients.

### Follow-up and recurrence treatment

After surgery, patients were followed up at regular intervals. Serum CEA and imaging studies were performed to detect any intrahepatic recurrence or distant metastases. The first follow-up occurred one month post-surgery, with subsequent ones every 3 months for up to 2 years, and every 6 months thereafter.

If tumor recurrence occurred and all recurrence sites were deemed treatable with salvage therapy, then active treatment was undertaken. Neither the number of metastases nor the recurrence site alone excluded any patients from salvage treatment. Otherwise, systematic chemotherapy was applied.

### Statistical analyses

Continuous variables are expressed as median (range). Continuous and categorical variables were compared using Mann–Whitney *U* test and χ^2^ test respectively. Multivariate logistic regression analysis was performed by the backward elimination method to identify independent factors associated with early recurrence. The Hosmer-Lemeshow test was used to assess the fit of the logistic regression model. A scoring system that predict early recurrence was generated using all factors that had *P* < 0.05 according to the multivariate regression model. The discrimination ability of the scoring system was evaluated using Harrell's concordance index. The internal validation of the discrimination ability of the scoring system was performed using the bootstrapping techniques. Survival analyses were done using the Kaplan-Meier method, with comparisons by means of the log rank test. Multivariate models were constructed for OS using the Cox proportional hazard method. Variables were included in each multivariate model if they achieved a *P* < 0.1 for significance on univariate analysis. *P* < 0.05 was considered to indicate statistical significance. Statistical analyses were performed using the SPSS (version 22, Armonk NV, USA) and R software (http://www.r-project.org).

## CONCLUSIONS

Early recurrence within 6 months after hepatectomy for CRLM occurs in roughly one-thirds of patients and correlates with a poor prognosis. The independent risk factor for early recurrence are preoperative CEA ≥ 30 ng/ml, primary tumor LVI, the number of metastases ≥ 4, R1 resection and initially unresectable disease. Salvage treatment after early recurrence increases the chance of long-term survival in selected patients. Nevertheless, it does not offer a therapeutic benefit for high-risk patients (three or more risk factors). Based on these information, we developed a predictive model which differentiates patients at high risk of early recurrence from those at low risk, and will also reduce the inclusion of patients for unnecessary treatment after early recurrence.
